# Diallyl Trisulfide Enhances the Survival of Multiterritory Perforator Skin Flaps

**DOI:** 10.3389/fphar.2022.809034

**Published:** 2022-02-15

**Authors:** Chengji Dong, Zhuliu Chen, Linxin Zhu, Najeeb Bsoul, Hongqiang Wu, Jingtao Jiang, Xuankuai Chen, Yingying Lai, Gaoxiang Yu, Yanlan Gu, Xiaoshan Guo, Weiyang Gao

**Affiliations:** ^1^ Department of Orthopaedics, The Second Affiliated Hospital and Yuying Children’s Hospital of Wenzhou Medical University, Wenzhou, China; ^2^ Zhejiang Provincial Key Laboratory of Orthopaedics, Wenzhou, China; ^3^ The Second Clinical Medical College of Wenzhou Medical University, Wenzhou, China; ^4^ Department of Histology and Embryology, Wenzhou Medical University, Zhejiang, China

**Keywords:** multiterritory perforator flap, diallyl trisulfide, angiogenesis, autophagy, apoptosis, oxidative stress

## Abstract

The multiterritory perforator flap is one of the widest flap patterns used to repair tissue defects. However, flap necrosis of the distal part is still a challenging issue for plastic surgeons. Diallyl trisulfide (DATS) is an efficient ingredient extracted from garlic, exerting many important effects on different diseases. Our experiment aims to reveal whether DATS has a beneficial effect on the survival of perforator flaps and to explore its mechanism of action. The results showed that DATS enhanced angiogenesis and autophagy and reduced cell apoptosis and oxidative stress, thereby improving the survival rate of skin flaps. After co-administration with autophagy inhibitor 3-methyladenine (3MA), perforator flap survival was further improved. Mechanistically, we showed that PI3K/Akt and AMPK-HIF-1α signaling pathways in flap were activated under DATS treatment. All in all, DATS promoted the survival of multiterritory perforator flaps *via* the synergistic regulation of PI3K/Akt and AMPK-HIF-1α signaling pathways, and inhibition of DATS-induced autophagy further improves flap survival.

## Introduction

The multiterritory perforator flap is an extensive surgical technique for filling skin defects from skin disease, including trauma, tumor ablation, and complications of diabetes (Behan, 2003; Geddes et al., 2003). However, necrosis at the dynamic territory boundary and at the potential territory bothers reconstructive surgeons, which drastically limits its clinical application.

Choke vessels are resistant vessels that link adjacent vascular territories (Zhuang et al., 2012). When the multiterritory perforator flap is established, the blood supply at the distal of the flap is interrupted due to the closure of choke vessels in the second choke zone, and the distal portion of the flap is under the condition of ischemia and hypoxia (Wang et al., 2017a). Subsequently, due to the opening of choke vessels in the choke II zone, the distal blood flow of the flap recovers, which will also lead to ischemia–reperfusion (I/R) injury of the flap (Siemionow and Arslan, 2004; Wang et al., 2011). Ischemia and I/R injury can lead to a burst of reactive oxygen species (ROS) generation and cell apoptosis, ultimately resulting in distal necrosis of the multiterritory perforator flap. Hence, intervening angiogenesis, oxidative stress, and cell apoptosis are considered to be effective therapeutic strategies for enhancing multiterritory perforator flap survival (Xin et al., 2020).

I/R injury plays an important role in the survival of skin flaps. I/R injury is divided into two processes (Soares et al., 2019). First, ischemia causes tissue ischemic supply leading to necrosis, and then reperfusion causes a series of reactions, including inducing a large amount of Ca^2+^ influx to cause Ca^2+^ overload, a large number of oxygen free radicals to increase accumulation, and the accumulation of neutrophils (Oliveira et al., 2018; Lv et al., 2021). These reactions induce cell apoptosis, iron death, etc. (Yan et al., 2020; Pan et al., 2021), further aggravating tissue necrosis, leading to necrosis of the distal skin flap.

Diallyl trisulfide (DATS), one of the major and biologically active components of garlic oil, exhibits a beneficial effect on the recovery of I/R injury. For example, it is suggested that DATS could be used for the treatment of I/R injury in brain tissues for its inhibitory effects on oxidative stress through Nrf2 activation (Silva-Islas et al., 2019). Besides, DATS is also believed to own the ability for the promotion of angiogenesis (Hayashida et al., 2017), which can be utilized for the protection of ischemic tissue in diabetic mice (Yang et al., 2018) as well as the salvation of the ischemic limb (Hsieh et al., 2019). In addition, there is a report suggesting that DATS is involved in the interruption of cell apoptosis through the induction of autophagy (Chu et al., 2012). Despite its significant therapeutic benefits in many disease models of I/R injury (Jeremic et al., 2019; Silva-Islas et al., 2019), whether DATS can play a therapeutic role in flap survival is completely unknown.

Autophagy has many physiological and pathophysiological roles in a variety of diseases (Mizushima, 2007). For example, increasing autophagy can reduce steroid-induced apoptosis of bone cells (Yue et al., 2021) and protect cells from premature aging (Rossiter et al., 2021). In addition, activation of FUNDC1-dependent mitophagy protects neurons against cerebral I/R injury (Cai et al., 2021). However, autophagy may have adverse effects on the progression of the disease (Wu et al., 2018; Sun et al., 2020). A recent study demonstrated that the circular RNA ACR attenuates autophagy and cell death in cardiomyocytes *via* modulation of the Pink1/FAM65 pathway (Zhou L. Y. et al., 2019). Moreover, MiR-181b plays a protective role in KA-induced epileptic juvenile rats by attenuating autophagy and apoptosis (Wang et al., 2019). Therefore, autophagy is a double-edged sword and may play different roles in different diseases and disease progress (Wang et al., 2021). Our previous studies have also shown that the effect of autophagy on flap viability is controversial and needs further research (Li et al., 2020; Zhang et al., 2020; Chen et al., 2021; Zhu et al., 2021; Lou et al., 2022). Therefore, the present study seeks to explore the effects of DATS and DATS-induced autophagy on the survival of perforator flaps and to reveal its possible regulatory mechanisms.

## Materials and Methods

### Reagents

DATS (C₆H₁₀S₃, purity ≥ 98%, Supplementary Figure S1A) was purchased from MCE (Monmouth Junction, NJ, USA; HY-117235), and 3-methyladenine (3MA) was acquired from Sigma-Aldrich Chemical Company (Milwaukee, WI, USA).

### Animals

One hundred eight male Sprague Dawley rats aging 8 weeks (weight, 250–300 g) were obtained from Animal Experiment Center at Wenzhou Medical University (license no. SYXK [ZJ]2020-0014). All animals were treated with humane care, and the protocol applied for animal research was in compliance with the ethical guidelines of the Laboratory Animals of China National Institutes of Health for animal experimentation. The experimental rats used in our work were permitted by the Animal Research Committee of Wenzhou Medical University (wydw 2021-0256). All rats were separated and then housed in a single cage with a 12-h light/dark cycle at an appropriate temperature of 23°C ± 2°C and humidity of 55% ± 10%, supplied with adequate amounts of food and water.

### Animal Model

**FIGURE 1:**
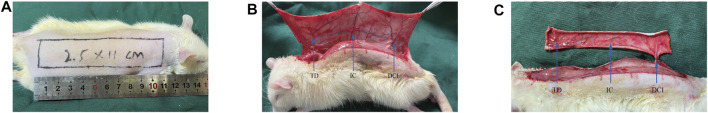
Multiterritory perforator skin flap model. **(A)** Scope of flap establishment. **(B)** The three main arteries are deep circumflex (DCI), thoracodorsal (TD), and posterior intercostal (IC). **(C)** IC and TD were disconnected, and DCI was preserved.

Before the operation, the rat dorsal midline was used as the medial border of the flap. The pedicle was located on the unilateral deep circumflex iliac (DCI) artery to establish a right-side perforator flap (2.5 × 11 cm^2^) in every rat as reported previously (Chen et al., 2020) (Figure 1A). After the flaps were completely lifted from the underlying fascia, a satisfactory hemostatic effect was obtained. Three vascular territories were defined: DCI area, intercostal (IC) area, and thoracodorsal (TD) area (Figure 1B). DCI was retained, while the other two vessels were ligated (Figure 1C). Then the flap was sutured *in situ* with 4–0 silk.

### Drug Administration

DATS was dissolved in dimethyl sulfoxide (DMSO) and diluted with corn oil stored at −20°C. To determine the appropriate oral dose of DATS, thirty rats were randomly divided into the five groups after surgery (*n* = 6) and received DATS orally for 7 consecutive days with doses of 0, 15, 30, 45, and 60 mg/kg. After the determination of the appropriate DATS dose, animals were randomly divided into three groups, as follows: 1) the experimental group (*n* = 24) received DATS orally (30 mg/kg/day) for 7 consecutive days, 2) the control group (*n* = 24) received an equal volume of DMSO-oil solution simultaneously, and 3) the DATS+3MA group (*n* = 30) received 3MA (10 mg/kg/day) 30 min before DATS treatment (Jin et al., 2018). All animals were euthanized after the experiment. Injection processes were achieved by one experimenter in order to reduce potential experimental error.

### Flap Survival Measurement

On postoperative days 3 and 7, observations of the flap survival status were recorded by high-quality photographs using a digital camera. Image-Pro Plus imaging software (ver. 6.0; Media Cybernetics, Rockville, MD, USA) was applied to assess flap viability. The surviving area was calculated by the following formula (viable area size/full flap size) × 100.

### Laser Doppler Imaging

A laser Doppler imager (Moor Instruments, Axminster, UK) was applied to measure hemoperfusion in the flap on days 3 and 7. The color-coded living flux images were obtained after scanning the dorsal flap of rats, which were fully anesthetized. The images were deposited in moor LDI Review software (ver.6.1; Moor Instruments) to quantify hemoperfusion of perforator flaps.

### Histological Examination

Equal-size specimens (*n* = 6, 0.5 × 0.5 cm^2^) were gathered from the middle part of the second choke vessel (SCV) zone in each group after animals were euthanized, then fixed in 4% paraformaldehyde for 1 day, and implanted in paraffin wax. Subsequently, every specimen was cut into tissue sections (4 µm thickness). The microscopic state of flap tissues was assessed by using a light microscope (Nikon, Tokyo, Japan). Every sample randomly selected six fields to observe angiogenesis. Then, the level of microvascular density (MVD) environment was quantified by calculating the number of vascular cross section per unit area (/mm^2^).

### Immunohistochemical Staining

The processed samples mentioned above were then deparaffinized in xylene, and different concentrations from 100% to 75% of ethanol baths were applied for rehydration. Afterward, the rehydrated specimens were immersed in 3% (v/v) hydrogen peroxide to block endogenous peroxide activities and kept in 10.2 mM of sodium citrate for antigen repair at 95°C for 20 min. Finally, samples were incubated overnight at 4°C with the following required primary antibodies, including anti-Cadherin 5 (1:100, Affinity, Cincinnati, OH, USA), anti-CD34 (1:100, Abcam, Cambridge, UK), anti-VEGF (1:300, Abcam), cleaved caspase-3 (1:200, Cell Signaling Technology, Danvers, MA, USA), anti-SOD1 (1:100, ProteinTech, Chicago, IL, USA), or anti-CTSD (1:100, ABclonal, Woburn, MA, USA), then treated with horseradish peroxidase (HRP)-conjugated secondary antibody, and stained again with hematoxylin. The DP2-TWAN image-acquisition system (Nikon, Tokyo, Japan) was performed to acquire images of the tissue sections on the light microscopy at ×200 magnification. Images were analyzed to quantify for Cadherin 5, CD34, VEGF, C-CASP3, SOD1, and CTSD expression levels; and the amount of CD34-positive microvessels was enumerated. Assessments were acquired from six random visual fields in three tissue sections.

### Flap Angiography

On the 7th day after surgery, six anesthetized rats received whole-body angiography as reported previously (Jiang et al., 2020). Briefly, lead oxide-gelatin (80 ml/kg) was administered into the exposed right carotid artery *via* a 24-gauge intravenous catheter until the limbs turned yellow. After treatment, rats were stored at −80°C overnight, and then an X-ray machine (54 kVp, 40 mA, 100-s exposure) was applied to scan flap samples. The number of choke vessels was quantified to reflect the effect of DATS intervention.

### Immunofluorescence

We deparaffinized and rehydrated six specimens as described in the process for immunohistochemistry (IHC). After that, the tissue antigen was retrieved with sodium citrate buffer (10.2 mM). Then 0.1% (volume ratio) phosphate-buffered saline (PBS) Triton X-100 was used to permeate the sample; this was followed by blocking in 10% (v/v) goat serum for 1 h. Slides are incubated (at 4°C) with LC3II (1:200, ProteinTech) and α-SMA (1:200, ProteinTech) for 16 h. The second antibody was then incubated with the specimen at 27°C for 1 h. Under the fluorescence microscope (Nikon), six random areas in three sections of tissue samples were randomly selected for analysis to determine the number of α-SMA-positive microvessels and the positive cell rate of LC3II.

### Western Blotting Analysis

Western blotting analysis of proteins in flap tissue was conducted as described previously (Chen et al., 2020), including the following proteins: VEGF (1:1,000, ProteinTech), Cadherin 5 (1:1,000, Affinity), LC3II (1:500, ProteinTech), SOD1 (1:1,000, ProteinTech), C-CASP3 (1:1,000, CST), MMP9 (1:1,000, Abcam), SQSTM1/p62 (1:1,000, Abcam), Bax (1:1,000, ProteinTech), CTSD (1:1,000, ABclonal), HO-1 (1:1,000, ProteinTech), Bcl-2 (1:1,000, CST), eNOS (1:1,000, Abcam), VPS34 (1:1,000, ProteinTech), Beclin1 (1:1,000, ProteinTech), PI3K (1:1,000, ProteinTech), AKT (1:1,000, ProteinTech), p-PI3K (1:1,000, CST), *p*-Akt (1:1,000, CST), AMPK (1:1,000, CST), *p*-AMPK (1:1,000, CST), HIF-1α (1:1,000, Abcam), and GAPDH (1:1,000, ProteinTech). Finally, the intensity of Western blotting was quantified using Image Lab 3.0 software (Bio-Rad, Hercules, CA, USA).

### Measurement of Glutathione and Malondialdehyde Levels

Malondialdehyde (MDA) test kits acquired from Beyotime Biotechnology (Shanghai, China) were used to evaluate the oxidative stress of the skin flap. Glutathione (GSH) test kits were acquired from NanJing JianCheng Bioengineering Institute (Nanjing, China). Six samples from the SCV zone followed the reagent instructions.

### Statistical Analyses

The statistical analysis of experimental data was executed *via* SPSS version 23. All data are indicated as means ± standard error. Independent-samples *t*-test was used to compare the mean difference between the two groups. *p* < 0.05 was regarded as statistically significant.

## Results

### Diallyl Trisulfide and 3-Methyladenine Enhance Multiterritory Perforator Flap Survival

**FIGURE 2:**
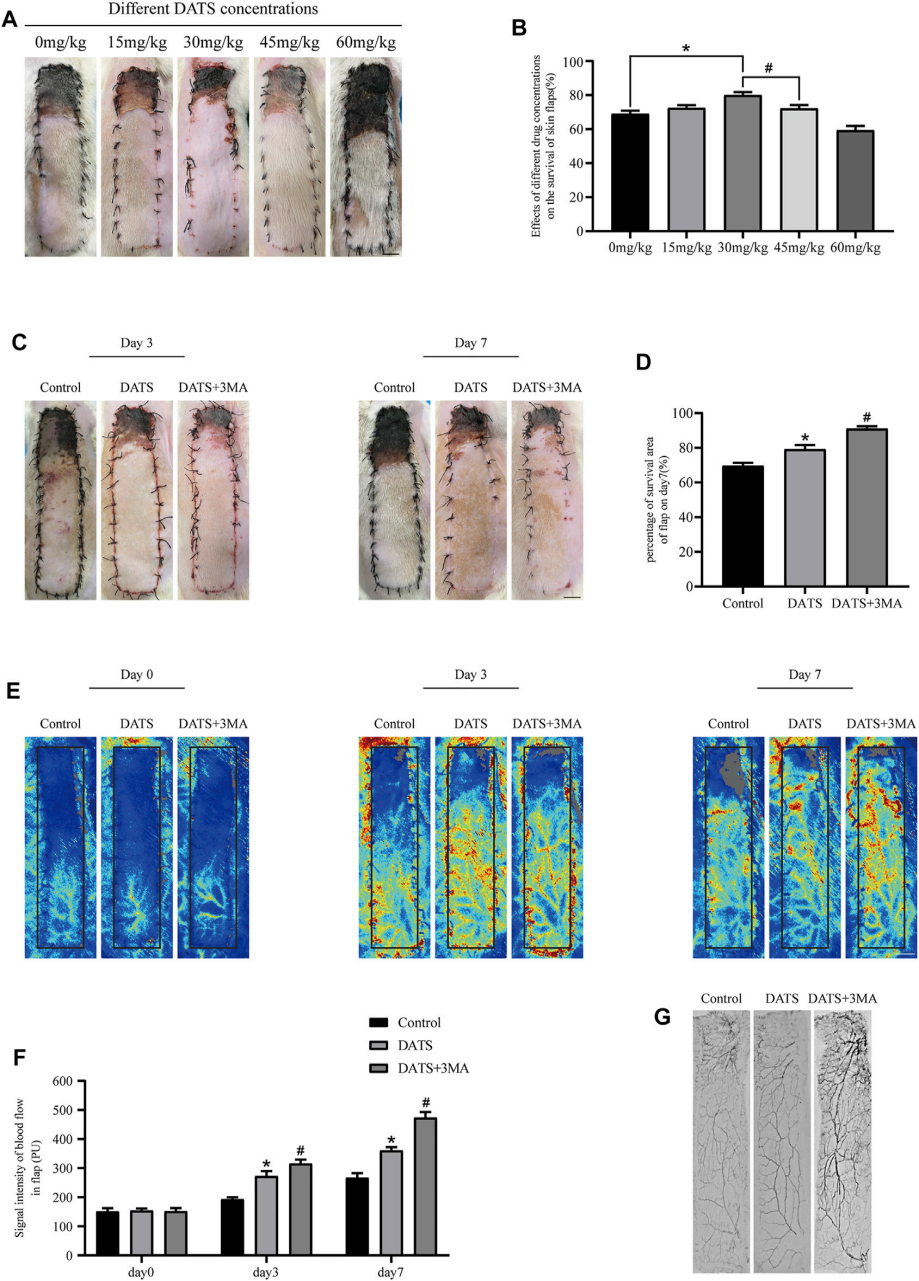
DATS and 3MA ameliorate the survival of multiterritory perforator flap. **(A)** Effects of different doses of DATS on the survival area on POD 7. **(B)** Statistical chart of survival area of skin flap treated with different doses on POD 7. **(C)** Digital high-definition photos of flap survival/necrosis area in the control, DATS, and DATS+3MA groups on the 3rd and 7th PODs. **(D)** Statistical chart of survival area of skin flap in each group. **(E)** LDBF in a perforator flap in the control, DATS, and DATS+3MA groups on the 0th, 3rd, and 7th PODs. **(F)** Statistical chart of the quantified blood flow signal intensity in each group on the 0th, 3rd, and 7th PODs. **(G)** Flap angiograms in each group on the 7th POD. *p < 0.05, vs. control group; #*p* < 0.05, vs. DATS group. The obtained data were expressed as means ± SEM, *n* = 6 for every group. DATS, diallyl trisulfide; 3MA, 3-methyladenine; POD, postoperative day; LDBF, laser Doppler blood flow.

First, thirty rats were administered different doses of DATS to determine the appropriate dose for flap survival (Figure 2A). As seen in Figure 2B, the survival area of the flap increased with the increase of DATS doses from 0 to 30 mg/kg, whereas it decreased with an administered dose of DATS from 45 to 60 mg/kg. Combined with the results of blood flow (Supplementary Figures S1B,C), 30 mg/kg was determined as the best dose for flap survival and was used as the experimental dose in subsequent experiments. After the operation, the skin was blackened, and edema and necrosis gradually developed from the top of the flap to the pedicle and then stabilized on the 7th day of operation (Figure 2C). The survival area of the DATS group was more than that of the control group, and the survival area increased after 3MA was added (Figure 2D). We used laser Doppler to analyze the blood flow signal and the formation of the microvascular network (Figure 2E). The results showed that there was no blood flow signal difference between the three groups after the operation. On the third and seventh days after the operation, the blood flow signals of the DATS+3MA group were higher than those in the DATS group and control group, among which the blood flow signals of the control group were the lowest (Figure 2F). The results showed that the distal vessels of the DATS+3MA group could be well filled by lead oxide angiography, the distal part of the DATS group was filled, and the distal part of the control group was lumpy or even absent (Figure 2G). In general, these data suggest that DATS can increase blood flow, promote flap survival, and further enhance these effects by combining with 3MA.

### Diallyl Trisulfide Induces Autophagy in the Multiterritory Perforator Flap

**FIGURE 3:**
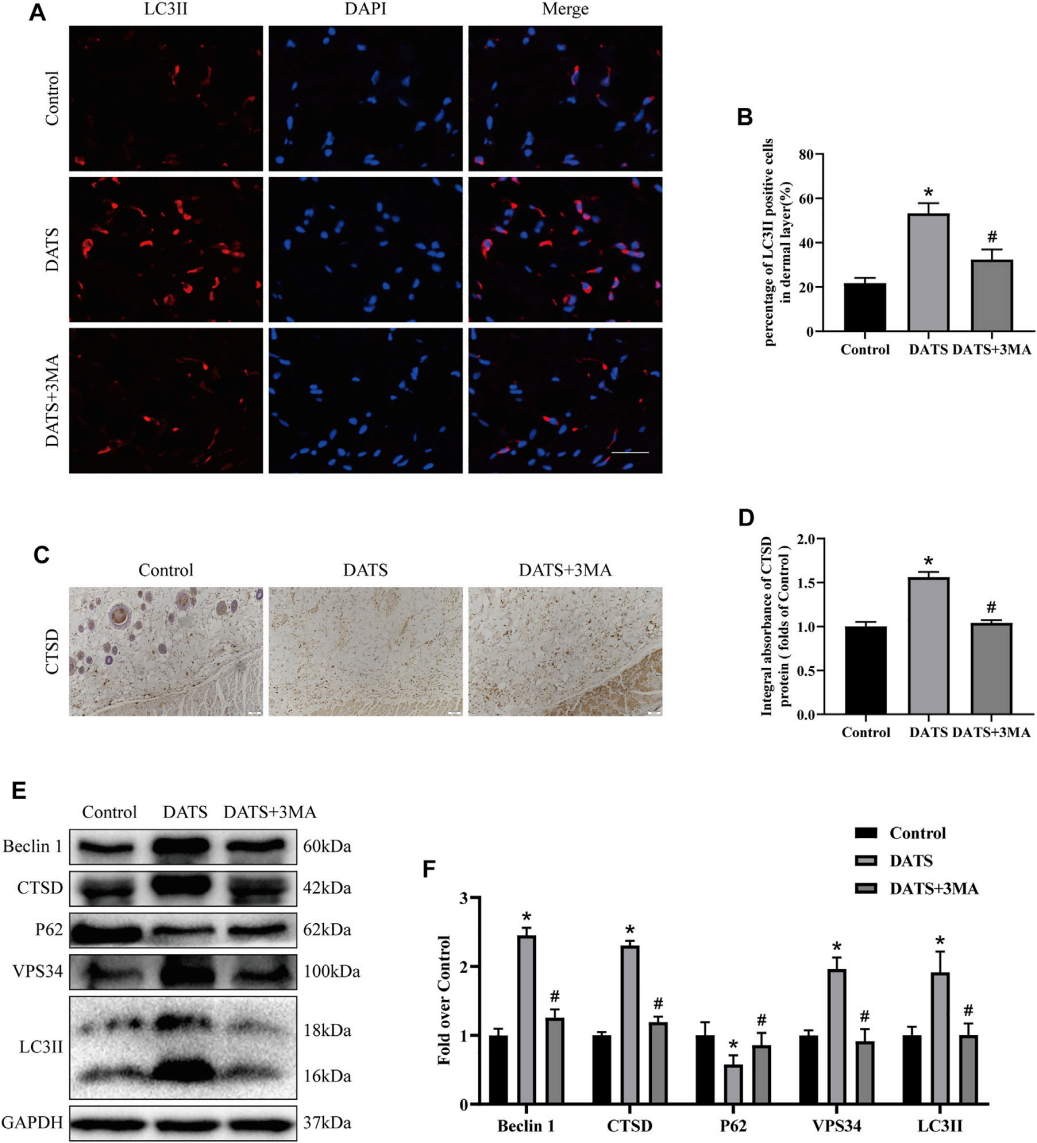
DATS induces autophagy in the multiterritory perforator flap. **(A)** Autophagosomes (LCII, red) and nuclei (DAPI, blue) in cells in the choke II zone of flaps in the control, DATS, and DATS+3MA groups (scale bar: 50 µm). **(B)** Statistical chart of the percentage of the LC3-positive cells. **(C)** IHC for CTSD in the multiterritory perforator flap in each group (original magnification ×200; scale bar, 50 µm). **(D)** Statistical chart of the CTSD expression level estimated by IHC. **(E)** Western blotting of autophagy markers, Beclin1, CTSD, LC3II, VPS34, and p62 in the control, DATS, and DATS+3MA groups. All the gel electrophoresis experiments were carried out under the same experimental conditions. **(F)** Statistical chart of the quantification of Beclin1, CTSD, LC3II, VPS34, and p62 expressions in the perforator flaps. *p < 0.05, vs. control group; #*p* < 0.05, vs. DATS group. The obtained data were expressed as means ± SEM, *n* = 6 for every group. DATS, diallyl trisulfide; 3MA, 3-methyladenine; IHC, immunohistochemistry.

We explored the role of autophagy in the perforator flap by detecting the expression of autophagy-related proteins in tissues. In this study, we mainly analyzed auto-phagosomal proteins (VPS34, Beclin1, and LC3II), lysosome protein (CTSD), and autophagic substrate protein (SQSTM1/p62). In the results of immunofluorescence, we found that the rate of LC3-positive cells in the DATS group was higher than that in the control group (Figures 3A,B). After that, we analyzed the integral absorbance of CTSD in IHC and found that the DATS group was higher than the control group (Figures 3C,D). Western blotting showed that the levels of Beclin1, VPS34, CTSD, and LC3II in the DATS group were higher than those in the control group, but the level of p62 was lower (Figure 3E). After 3MA was added to the DATS group, we analyzed the autophagy-related protein expression in the DATS group and DATS+3MA group. In immunofluorescence, the ratio of LCII-positive cells in DATS+3MA was lower than that in the DATS group, which was consistent with the results obtained in Western blotting, and the integral absorbance of CTSD in the DATS+3MA group was lower than that in the DATS group in IHC (Figure 3F). Further analysis of Western blotting showed that the expressions of Beclin1, VPS34, CTSD, and in the DATS+3MA group were lower than those in the DATS group, while p62 was higher than that in the DATS group, which verified that 3MA reversed the autophagy induced by DATS. According to the above results, we found that DATS can induce autophagy and that 3MA can inhibit autophagy, which is induced by DATS.

### Diallyl Trisulfide Affects Angiogenesis in the Multiterritory Perforator Flap

**FIGURE 4:**
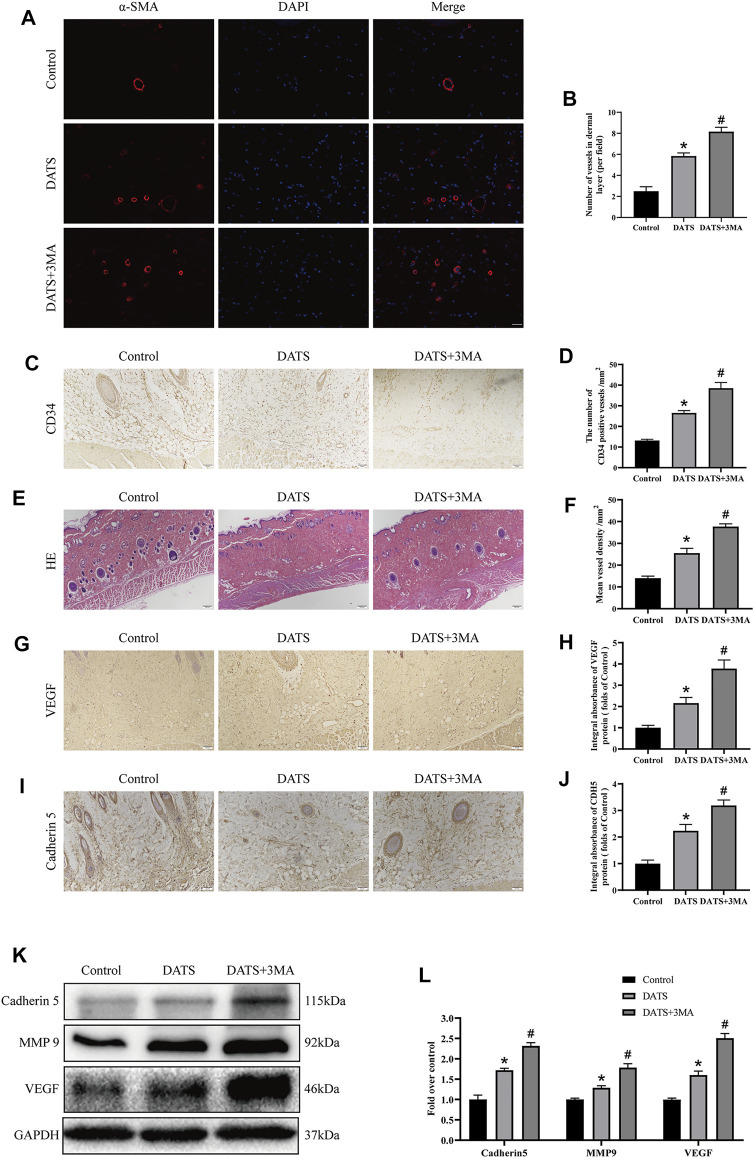
DATS-induced autophagy affects angiogenesis in the multiterritory perforator flap. **(A)** α-SMA-positive microvessels in each group were evaluated by immunofluorescence (scale bar: 50 µm). **(B)** Statistical chart of the number of α-SMA marked microvessels in each group. **(C)** IHC for CD34-positive microvessels in each group (original magnification ×200; scale bar, 50 µm). **(D)** The statistical chart describes the CD34-positive vessel density. **(E)** The H&E staining shows the microvessels and subcutaneous histology in each group (original magnification ×100; scale bar, 100 μm). **(F)** The statistical chart describes the percentage of microvascular density (MVD). **(G, I)** IHC for Cadherin 5 and VEGF in the multiterritory perforator flap in each group (original magnification ×200; scale bar, 50 µm). **(H, J)** Statistical chart of the Cadherin 5 and VEGF expression levels estimated by IHC. **(K)** Western blotting of angiogenesis markers, Cadherin 5, VEGF, and MMP9 in the control, DATS, and DATS+3MA groups. All the gel electrophoresis experiments were carried out under the same experimental conditions. **(L)** Statistical chart of the quantification of Cadherin 5, VEGF, and MMP9 expressions in the perforator flaps. *p < 0.05, vs. control group; #*p* < 0.05, vs. DATS group. The obtained data were expressed as means ± SEM, *n* = 6 for every group. DATS, diallyl trisulfide; IHC, immunohistochemistry.

Angiogenesis is essential to improve the survival of the multiterritory perforator flap. We studied the mechanism of DATS-induced autophagy in angiogenesis according to the indicators of angiogenesis. In immunofluorescence, we detected the microvessel expression of α-SMA (Figure 4A). The number of microvessels in the control group was the least, and the number of microvessels in the DATS+3MA group was the highest among the three groups (Figure 4B). We also found the same situation in CD34-positive microvessels in IHC and H&E (Figures 4C,E); that is, the number of microvessels in the DATS and DATS+3MA groups was higher than that in the control group, and the number of microvessels in the DATS+3MA group was the highest among the three groups (Figures 4D,F). By detecting VEGF and Cadherin 5 in IHC and Western blotting (Figures 4G,I), we found that the control group was at the lowest level, the DATS group was higher than the control group, and the DATS+3MA group had the highest expression (Figures 4H,J). In addition, the expression of MMP9 was the highest in the DATS+3MA group and the lowest in the control group (Figures 4K,L). These results suggest that angiogenesis in skin flap was enhanced by DATS and was further promoted after autophagy inhibition by 3MA.

### Diallyl Trisulfide Affects Oxidative Stress in the Multiterritory Perforator Flap

**FIGURE 5:**
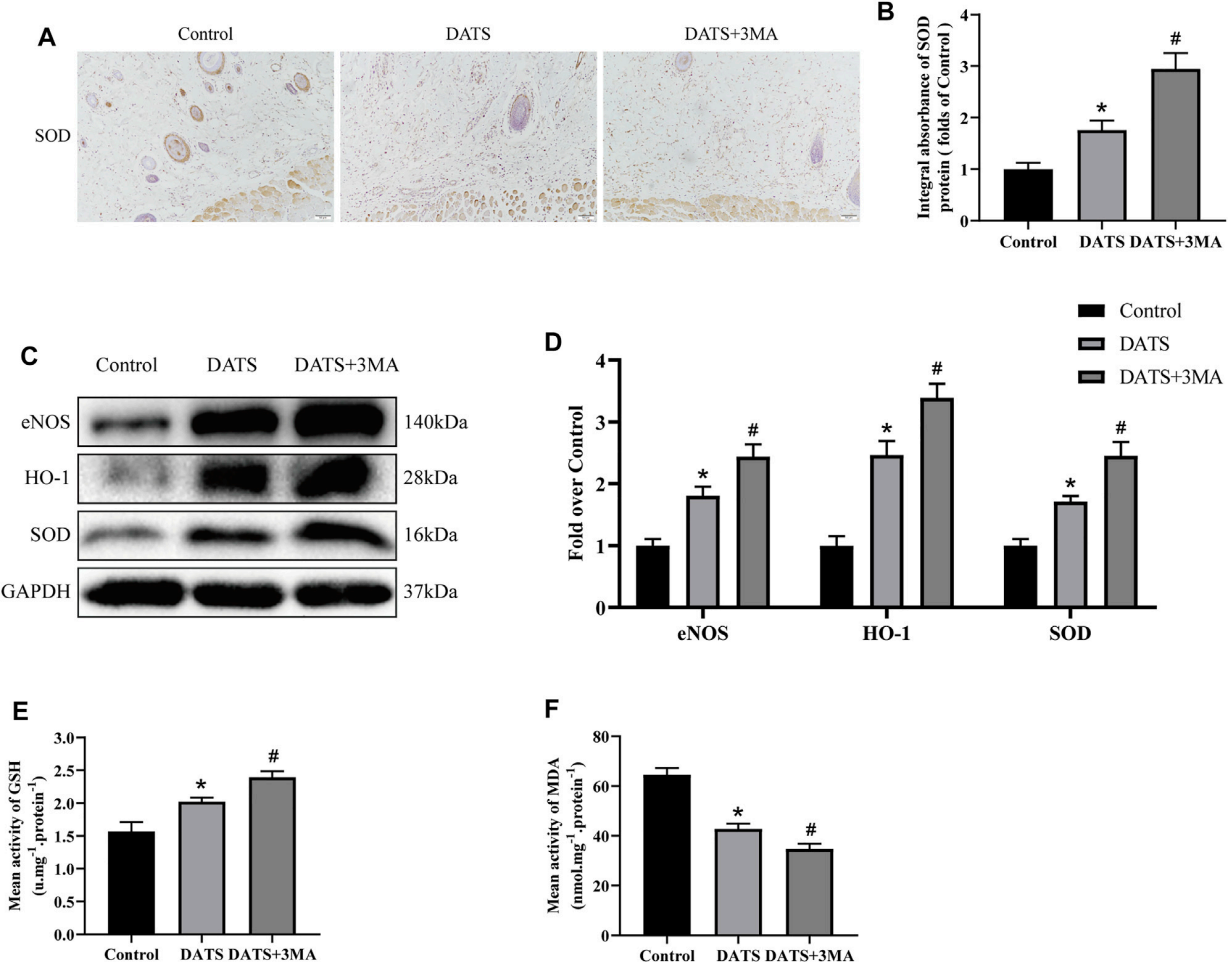
DATS-induced autophagy affects oxidative stress in the multiterritory perforator flap. **(A)** IHC for SOD1 in the multiterritory perforator flap in each group (original magnification ×200; scale bar, 50 μm). **(B)** Statistical chart of the SOD1 expression level estimated by IHC. **(C)** Western blotting of oxidative stress markers, SOD1, HO-1, and eNOS in the control, DATS, and DATS+3MA groups. All the gel electrophoresis experiments were carried out under the same experimental conditions. **(D)** Statistical chart of the quantification of SOD1, HO-1, and eNOS expressions in the perforator flaps. **(E)** Statistical chart of the quantification of GSH. **(F)** Statistical chart of the quantification of MDA. *p < 0.05, vs. control group; #*p* < 0.05, vs. DATS group. The obtained data were expressed as means ± SEM, *n* = 6 for every group. DATS, diallyl trisulfide; IHC, immunohistochemistry; GSH, glutathione; MDA, malondialdehyde.

Oxidative stress also plays an important role in multiterritory perforator flap survival. Therefore, we analyzed the expression of SOD1 in IHC (Figure 5A) and found that the integral absorbance of SOD1 in the DATS+3MA group was higher than that in the DATS group, and the control group was the lowest among the three groups (Figure 5B). In addition, we also analyzed the expression levels of HO-1, SOD1, and eNOS in the multiterritory perforator flap by Western blotting (Figure 5C). Compared with the control group, the protein expression level in the DATS group was higher, and the protein expression level of the DATS+3MA group was higher than that in the DATS group (Figure 5D). Similar results appeared in the content of GSH, and the opposite result appeared in the content of MDA (Figures 5E,F). From the above results, we found that DATS can reduce oxidative stress and significantly reduce oxidative stress after inhibiting autophagy induced by DATS.

### Diallyl Trisulfide Affects Apoptosis in the Multiterritory Perforator Flap

**FIGURE 6:**
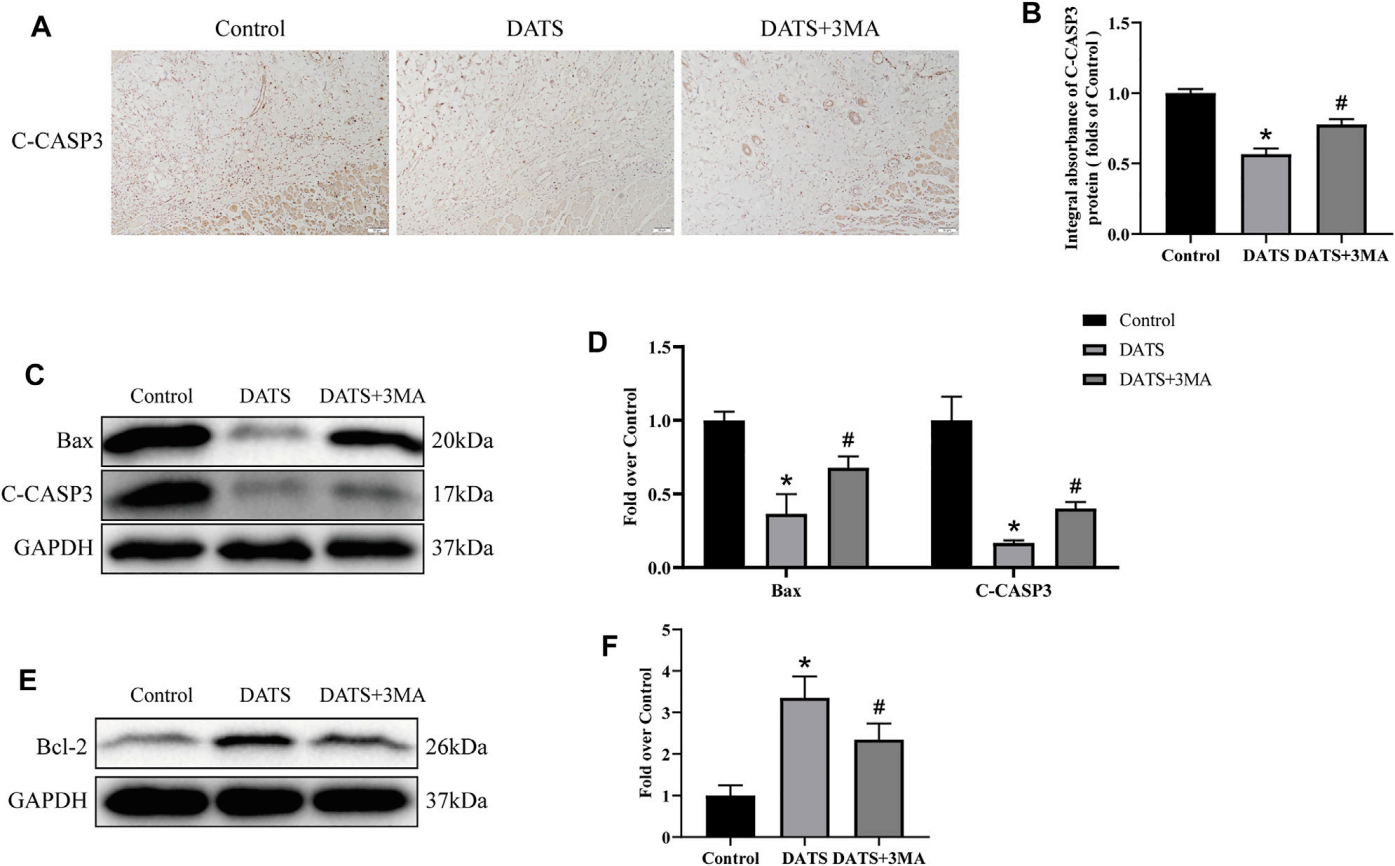
DATS-induced autophagy affects apoptosis in the multiterritory perforator flap. **(A)** IHC for C-CASP-3 in the multiterritory perforator flap in each group (original magnification ×200; scale bar, 50 μm). **(B)** Statistical chart of the C-CASP-3 expression level estimated by IHC. **(C)** Western blotting of oxidative stress markers, C-CASP-3, and Bax in the control, DATS, and DATS+3MA groups. **(D)** Statistical chart of the quantification of C-CASP-3 and Bax expressions in the perforator flaps. **(E)** Western blotting of oxidative stress markers and Bcl-2 in the control, DATS, and DATS+3MA groups. All the gel electrophoresis experiments were carried out under the same experimental conditions. **(F)** Statistical chart of the quantification of Bcl-2 expressions in the perforator flaps. *p < 0.05, vs. control group; #*p* < 0.05, vs. DATS group. The obtained data were expressed as means ± SEM, *n* = 6 for every group. DATS, diallyl trisulfide; IHC, immunohistochemistry.

Next, we study the effect of apoptosis-related protein in the multiterritory perforator flap. IHC and Western blotting analyses showed that the expression level of C-CASP-3 decreased in the DATS group, and the expression level of C-CASP-3 in the DATS+3MA group increased compared with the DATS group (Figures 6A,B). The expressions of Bax and C-CASP-3 in the three groups were consistent in Western blotting (Figures 6C,D). We found that the expression of Bcl-2 was the highest in the DATS group. The expression of Bcl-2 in the DATS+3MA group was lower than that in the DATS group but still higher than that in the control group (Figures 6E,F). So far, we found that DATS can reduce apoptosis and promote flap survival, while 3MA can reverse the active effect of autophagy induced by DATS.

### Diallyl Trisulfide Abates Apoptosis by the PI3K/Akt Signaling Pathway

**FIGURE 7:**
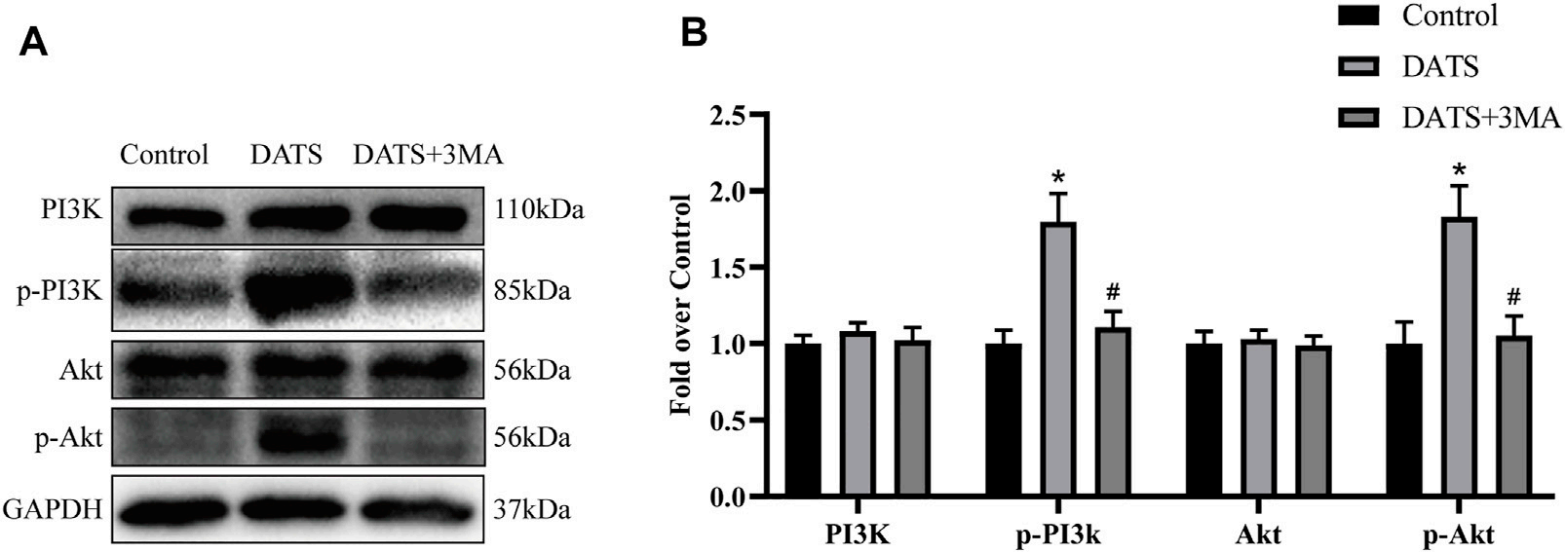
DATS abates apoptosis by the PI3K/Akt signaling pathway. **(A)** Western blotting of PI3K/Akt signaling pathway markers, PI3K, p-PI3K, Akt, and *p*-Akt in the control, DATS, and DATS+3MA groups. All the gel electrophoresis experiments were carried out under the same experimental conditions. **(B)** Statistical chart of the quantification of PI3K, p-PI3K, Akt, and *p*-Akt expressions in the perforator flaps. *p < 0.05, vs. control group; #*p* < 0.05, vs. DATS group. The obtained data were expressed as means ± SEM, *n* = 6 for every group. DATS, diallyl trisulfide.

We used Western blotting to analyze the protein expression of the PI3K/Akt pathway to determine whether DATS can reduce apoptosis through this pathway (Figure 7A). According to the results, we found that the expression levels of PI3K and Akt in the three groups were approximately equal, and there was no significant difference among the three groups. However, the amount of phosphorylated PI3K and Akt protein in the DATS group was higher than that in the control group and DATS+3MA group (Figure 7B). Our results show that DATS can regulate PI3K/Akt to reduce apoptosis in the multiterritory perforator flap, which can reasonably explain that the antiapoptotic effect of DATS is weakened after the addition of 3MA.

### Diallyl Trisulfide Promotes Angiogenesis and Reduces Oxidative Stress Through AMPK Pathway

**FIGURE 8:**
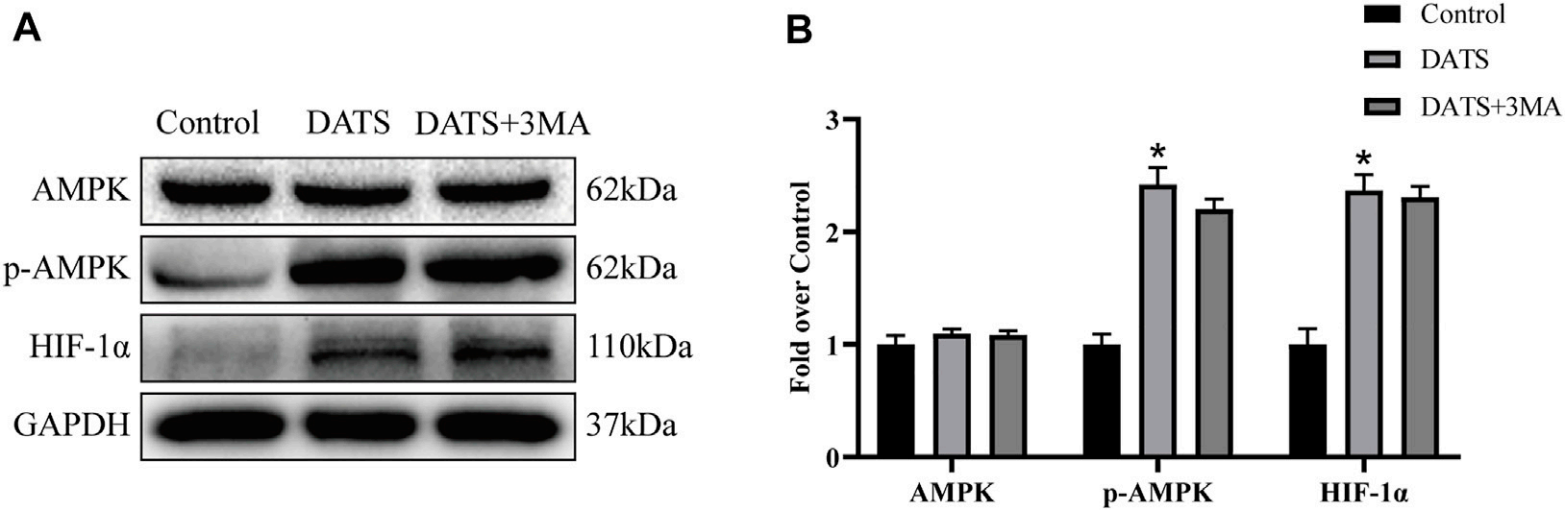
DATS promotes angiogenesis and reduces oxidative stress through the AMPK pathway. **(A)** Western blotting of AMPK signaling pathway markers, AMPK, *p*-AMPK, and HIF-1α in the control, DATS, and DATS+3MA groups. All the gel electrophoresis experiments were carried out under the same experimental conditions. **(B)** Statistical chart of the quantification of AMPK, *p*-AMPK, and HIF-1α expressions in the perforator flaps. *p < 0.05, vs. control group; #*p* < 0.05, vs. DATS group. The obtained data were expressed as means ± SEM, *n* = 6 for every group. DATS, diallyl trisulfide.

Next, we evaluated the expression of AMPK and HIF-1α. Our results showed that the expression levels of AMPK in the three groups were approximately equal. Besides, DATS increased the expression of *p*-AMPK and HIF-1α, and there was no significant difference between DATS and DATS+3MA (Figure 8A). We showed that DATS can upregulate AMPK and HIF-1α to promote angiogenesis and reduce oxidative stress in the multiterritory perforator flap.

## Discussion

Perforator flaps are widely applied for a variety of skin defects in plastic surgery (Kroll et al., 1988; Koshima et al., 1992; Geddes et al., 2003). However, perforator flap necrosis often occurs at the dynamic territory boundary and at the potential territory. To improve flap survival, strategies such as drug therapy, hyperbaric oxygen, delay procedure, and distal arterialized venous supercharging procedure were applied (Keleş et al., 2014; Wang et al., 2017b; Wu et al., 2021). Here, we investigated the potential benefits of DATS, a garlic organosulfur component, in a rat model with multiterritory perforator flaps. DATS has been shown to enhance blood flow recovery in the ischemic limbs *via* an endothelial nitric oxide synthase-dependent mechanism (Hayashida et al., 2017). Additionally, DATS effectively decreases acute ethanol-induced liver injury by attenuating oxidative stress and mitochondrial dysfunction (Zeng et al., 2008) and appears to inhibit cardiomyocyte apoptosis induced by high glucose through decreasing ROS generation (Kuo et al., 2013). However, few studies demonstrate the effect of DATS-induced autophagy on tissue survival (Liu et al., 2018). To the best of our knowledge, this is the first study to investigate the effect of autophagy induced by DATS on flap survival. Our results showed that the autophagy induced by DATS is harmful to the survival of the perforator flap, while 3MA exerts a positive effect on flap survival.

Autophagy is a self‐degradative process playing a key role in preventing various diseases (Glick et al., 2010; Jiao et al., 2021; Lee et al., 2021). Beclin1, LC3II, and VPS34 are important genes that participate in autophagosome formation (Yang and Klionsky, 2009), and p62 expression represents autophagosome degeneration after the fusion of autophagosomes with lysosomes, whereas CTSD is a characteristic of lysosomal function (Salazar et al., 2020). Nishida et al. demonstrated that either scarce or excessive autophagy was harmful (Nishida et al., 2008). Our results showed that DATS increased the expression of Beclin1, CTSD, VPS34, and LC3II while attenuating p62 expression. 3-MA is a well-known inhibitor of autophagy by blocking autophagy vesicles (Miller et al., 2010), which was used in different disease models to inhibit autophagy (Zhou K. et al., 2019; He et al., 2021; Li et al., 2022). However, after the addition of 3MA, the expression of Beclin1, CTSD, VPS34, and LC3II was decreased, while the expression of p62 was increased. Our experimental results confirmed that DATS could induce autophagy, but 3MA could reverse DATS-induced autophagy.

It is necessary to restore blood supply for flap timely and effectively. Therefore, the pro-angiogenesis ability of DATS treatment for the flap is evaluated. The dissociation of cell junctions is promoted by MMP9, which facilitates VEGF release to a certain degree (Li et al., 2018). The expression of extracellular proteases MMP9 and VEGF is associated with progressively intensifying angiogenesis (Giraudo et al., 2004). Cadherin 5, a cell-adhesion molecule, participates in the regulation of endothelial permeability and vascular integrity (Anderson et al., 2015). Our work found that the expression of MMP9, VEGF, and Cadherin 5 protein in the DATS-treated group was increased and that the protein expressions were further enhanced after co-administration with 3MA. Besides, we also observed the increase of MVD as well as more CD34-positive vascular cells, indicating that DATS improved angiogenesis of perforator flaps. These results were consistent with our angiography result that DATS was beneficial to the choke vessel’s dilation. At the same time, the blood flow signal was more obvious in the DATS+3MA group. Overall, DATS increased blood supply and promoted angiogenesis in the perforator flap, and these effects were strengthened after inhibition of DATS-induced autophagy by 3MA.

A previous study reported that IR injury happened in the potential territory of the perforator flap (Wang et al., 2017b). The accumulation of ROS induced by IR injury in ischemic tissue could cause cell death and tissue damage (Kim and Hong, 2007). Apoptosis is a classical programmed cell death, which is the main cell death in flap necrosis. This research has attempted to demonstrate the protective roles of DATS against oxidative stress and cell apoptosis for increasing perforator flap survival. A previous study revealed that DATS preserved cardiac function *via* decreasing oxidative stress and ER stress-mediated apoptosis (Yu et al., 2017a). SOD1 is an important enzyme against oxidative stress (Ferrari and Stagi, 2021), which can be used to evaluate the level of oxidative stress in skin flaps. In the current study, the proteins SOD1, eNOS, and HO-1 that exert antioxidative stress effects and the level of GSH were increased by DATS treatment in flap tissue, indicating that DATS has antioxidant properties for flap survival. As the expression of these antioxidative stress proteins increases, the level of MDA decreased accordingly. The antioxidative stress effect of DATS was enhanced after autophagy inhibition by 3MA. Moreover, the decrease of apoptosis-related proteins Bax, Bcl-2, and CASP3 expression indicated that DATS protected flap tissues from apoptosis. 3MA reduced the effects of DATS on antiapoptosis, but the perforator flap survival was enhanced.

As mentioned earlier, when 3MA was added to inhibit autophagy, cell apoptosis was increased, prompting us to study the mechanism of the interaction between autophagy and apoptosis. 3MA inhibitor is a selective PI3K inhibitor. When PI3K/Akt signaling pathway is activated, it can inhibit caspase activation during apoptosis and upregulate the activity of the Bcl-2 family proteins (New et al., 2007). Previous studies have shown that DATS can activate the PI3K/Akt pathway and reduce the apoptosis of human endothelial progenitor cells (Chiang et al., 2013). Our Western blotting results showed that p-PI3K and *p*-Akt were increased in the DATS group and were reversed by 3MA. We found that DATS can activate the PI3K/Akt pathway to reduce apoptosis, while 3MA inhibited the phosphorylation of PI3K and Akt, reversing the antiapoptotic effect of DATS.

Interestingly, apoptosis level was upregulated when autophagy was inhibited by 3MA, while the ability of angiogenesis and antioxidant stress was further enhanced, resulting in further survival of the flap. Previous studies have shown that DATS can protect cardiomyocytes from I/R injury through the AMPK pathway (Yu et al., 2017b). At the same time, the increased expression of AMPK and HIF-1α can promote angiogenesis and reduce oxidative stress (Choi et al., 2017; Zhao et al., 2021). Our results showed that *p*-AMPK and HIF-1α were increased in the DATS group and DATS+3MA group, and the difference between these two groups was not obvious. Consequently, DATS may enhance flap survival through the synergistic effect of PI3K/Akt and AMPK-HIF-1α signaling pathways. In other words, DATS regulated autophagy and apoptosis in perforator flaps mainly *via* PI3K/Akt signal pathway and regulated angiogenesis and oxidative stress mainly *via* the AMPK-HIF-1α signal pathway. Meanwhile, autophagy co-regulated angiogenesis and oxidative stress in perforator flaps. This explained why the ability of angiogenesis and antioxidant stress was further enhanced after autophagy was inhibited by 3MA. Of course, we need further research to demonstrate the relationship between these two signaling pathways.

There are still some limitations in our experiments. First, there is still a need for *in vitro* study for a better understanding of the mechanism referring to DATS-induced increase of autophagy. Second, more accurate regulation of autophagy is our next focus, by which autophagy may bring more benefits for the survival of perforator flap. Meanwhile, some studies reported that increasing autophagy can promote random flap survival (Anderson et al., 2015; Wu et al., 2019). The interpretation of this phenomenon may be correlated with the different sources of blood supply in these two models, leading to the different levels of autophagy, or the key regulatory protein is different, which need further experiments.

## Conclusion

In summary, we revealed that DATS increased the survival of multiterritory perforator flaps by enhancing angiogenesis and autophagy, alleviating oxidative stress and apoptosis. These effects were associated with the synergistic regulation of PI3K/Akt and AMPK-HIF-1α signaling pathways. Although inhibition of DATS-induced autophagy by 3MA weakened the antiapoptotic effect, the survival of skin flap was further promoted by increasing angiogenesis and antioxidant stress.

## Data Availability

The original contributions presented in the study are included in the article/Supplementary Material, further inquiries can be directed to the corresponding author.
