# Supplementary figures and images for: Diallyl Trisulfide Enhances the Survival of Multiterritory Perforator Skin Flaps
Source: Front Pharmacol. 2022 Feb 15;13:809034. doi: 10.3389/fphar.2022.809034 (PMC8885991; doi:10.3389/fphar.2022.809034)

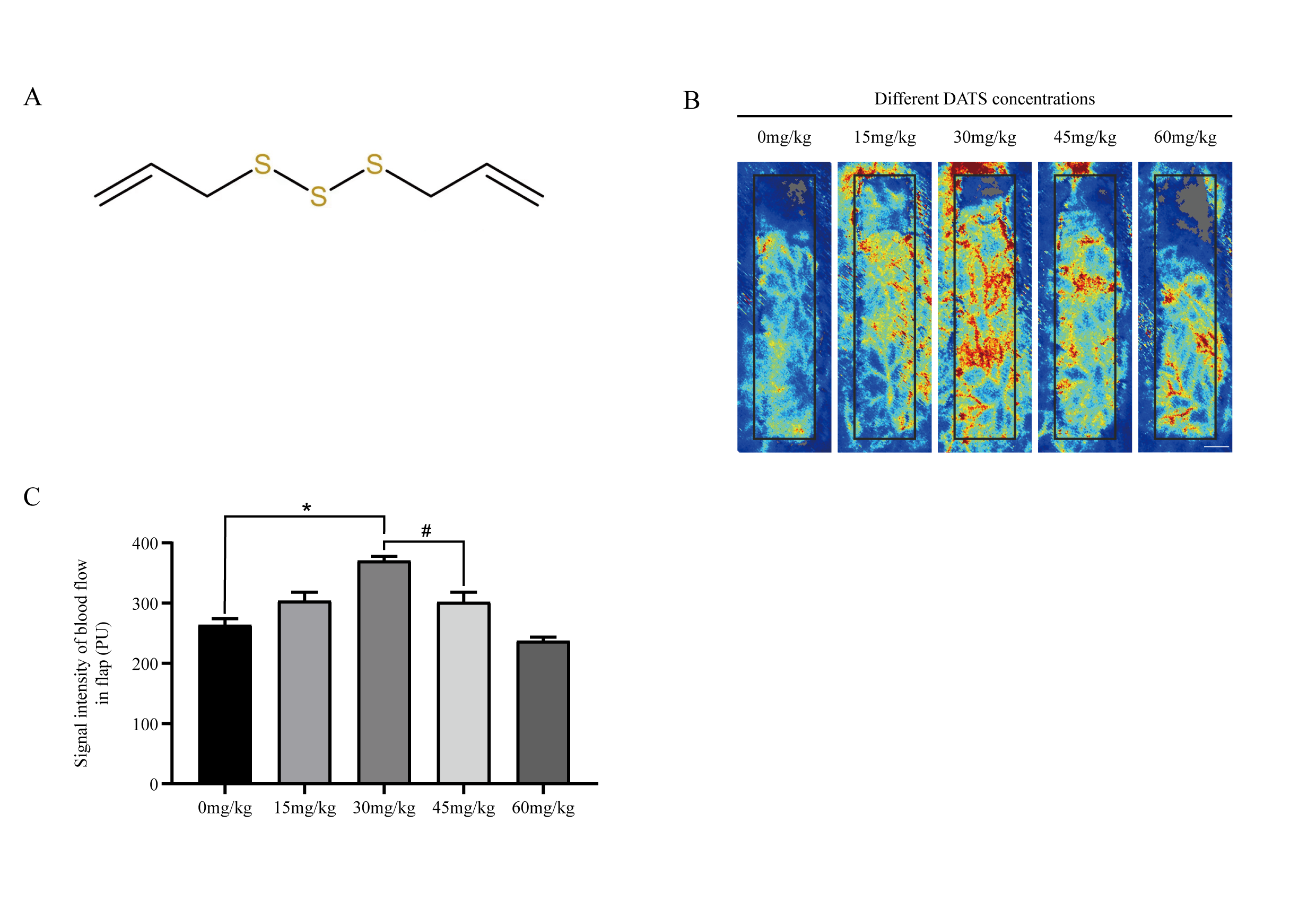

Supplement: Supplementary file 1 [file Image1.tif]
